# Papez Circuit Observed by *in vivo* Human Brain With 7.0T MRI Super-Resolution Track Density Imaging and Track Tracing

**DOI:** 10.3389/fnana.2019.00017

**Published:** 2019-02-18

**Authors:** Sang-Han Choi, Young-Bo Kim, Sun-Ha Paek, Zang-Hee Cho

**Affiliations:** ^1^Neuroscience Research Institute, Suwon University, Gyeonggi, South Korea; ^2^Neuroscience Research Institute, Gachon University, Incheon, South Korea; ^3^Department of Neurosurgery, Seoul National University Hospital, Seoul, South Korea; ^4^AICT, Seoul National University, Seoul, South Korea

**Keywords:** Papez circuit, perforant path, track density imaging, 7T DTI, fiber tracking

## Abstract

The Papez circuit has been considered as an important anatomical substrate involved in emotional experience. However, the circuit remains difficult to elucidate in the human brain due to the resolution limit of current neuroimaging modalities. In this article, for the first time, we report the direct visualization of the Papez circuit with 7-Tesla super-resolution magnetic resonance tractography. Two healthy, young male subjects (aged 30 and 35 years) were recruited as volunteers following the guidelines of the institutional review board (IRB). Track density imaging (TDI) generation with track tracing was performed using MRtrix software package. With these tools, we were able to visualize the entire Papez circuit. We believe this is the first study to visualize the complete loop of the Papez circuit, including the perforant path (PP), thalamocortical fibers of the anterior nucleus (AN), and mammillothalamic tract (MTT), which were hitherto difficult to visualize by conventional imaging techniques.

## Introduction

The classical Papez circuit is the neural loop goes through from hippocampal formation to mammillary body (MB) in the hypothalamus to anterior nucleus of the thalamus (AN) to cingulate gyrus/part of the parahippocampal gyrus (PHG) and back to the hippocampal formation. This loop provided for interaction among the neocortex, limbic structures and hypothalamus, and originally proposed that their interconnections might be the anatomical substrate of central emotion and emotional experience. The Papez circuit is now known to be more involved in the consolidation of declarative memory (Papez, 1937; John, 2009).

In more detail, the Papez circuit is composed of five neural paths: hippocampal formation—fornix—MB—mammillothalamic tract (MTT)—AN—thalamocortical fiber of the anterior nucleus (ANTCF)—cingulate gyrus—cingulum—PHG, entorhinal cortex (EC)—perforant path (PP), and back to the hippocampal formation (see Supplementary Figure S1; Augustinack et al., 2010). Although a number of non-invasive imaging studies have been conducted on the Papez circuit (Caldinelli et al., 2017; Wei et al., 2017), few imaging studies cover the entire Papez circuit in living humans. This is due to the inability to observe the Papez circuit in *in vivo* human, particularly the PP and the ANTCF (Choi et al., 2018).

Meanwhile, super-resolution track density imaging (TDI) has been developed and substantially improved the visualization of white matter structures (Calamante et al., 2013). The sensitivity of TDI is further improved when combined with ultra-high field 7-Tesla MRI (Calamante et al., 2013; Cho et al., 2015a). By using the post-processing methods to gain spatial resolution based on diffusion MRI fiber tracking, the super-resolution TDI can reveal structures beyond the resolution of the acquired imaging voxel (Calamante et al., 2010). Consequently, the 7-Tesla super-resolution TDI technique can now be used to analyze the fine structures of thalamocortical connections, such as the septum pallucidum tract (Cho et al., 2015a,b,c) and the ANTCF, the latter of which, as demonstrated earlier, is an important new component in the direct visualization of the Papez circuit (Choi et al., 2018).

In the present study, for the first time, we have delineated the entire structural connections of the Papez circuit using 7-Tesla TDI with the fiber tracking.

## Materials and Methods

There are three steps for the fiber visualization: acquisition of diffusion-weighted imaging (DWI) data, TDI data processing, and seed-based tracking analysis. For DWI data acquisition, a 7-Tesla MRI scanner (Magnetom 7.0T, Siemens, Erlangen, Germany) was used. Two healthy, young normal male subjects (aged 30 and 35 years) were recruited as volunteers following the recommendations of institutional review board (IRB) of Gachon Medical School and Korea Food and Drug Administration (KFDA) with written informed consent from all subjects. All subjects gave written informed consent in accordance with the Declaration of Helsinki. The protocol was approved by the IRB of Gachon Medical School and KFDA. DWI data was acquired using a single-shot echo-planar imaging sequence with the following parameters: repetition time/echo time = 6,000/83 ms; matrix size = 128 × 128 (field of view 230 mm × 45 slices); 1.8 mm isotropic resolution; 64 DWI directions; b-value = 2,000 s/mm^2^, with a *b* = 0 image; GRAPPA with factor 3; and a bandwidth of 1,562 Hz/px. Scans were performed in triplicate format with a total acquisition time of 19 min and 5 s.

DWI data were then processed using the TDI image processing technique (Calamante et al., 2013). TDI analysis was carried out using MRtrix software package (Brain Research Institute, Florey Neuroscience Institute, Melbourne, VIC, Australia). The relevant tracking parameters were: tracking type = SD-PROB; track minimum length = 20 mm; step-size = 0.02 mm; curvature radius constraint = 0.04 mm; fiber orientation distribution cutoff for track termination = 0.3; and number of tracks = 6,000,000 (Tournier et al., 2012). The final TDI image was generated with a nominal isotropic resolution of 0.18 mm.

**Figure 1:**
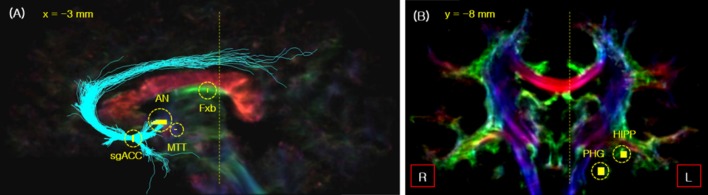
The seed_points used for fiber tracking of the Papez circuit in the sagittal **(A)** and coronal view **(B)**. The seed_points are indicated by short yellow lines with the yellow circles. The two vertical yellow dotted lines in images **(A,B)** indicate the slice cut of each image. As indicated, the sagittal slice is cut view at *x* = 3 mm, while the coronal slice is at *y* = −8 mm. Legend: Fxb, fornix body; HIPP, hippocampal formation.

Seed tracking analysis of the four paths of the Papez circuit (ANTCF, fornix, MTT, and EC area) were performed independently. The seed positions were located based on the super-resolution TDI image rather than the structural MRI anatomical image for the better positional accuracy (Figure 1). The structural MRI image shows a better boundary of the gray matter or vessel, however, super-resolution TDI image is markedly better in visualization of structure and contrast of the white matter area. Furthermore, based on structural MRI seed positioning approach, intrinsic positioning error between the DWI data cannot be avoided. Therefore, the super-resolution TDI image is better suited for the accurate seed positioning in the white matter fiber tracking (Calamante et al., 2013; Cho et al., 2015a).

First, the subgenual anterior cingulate cortex (sgACC) was selected as the seed position for the fiber tracking of ANTCF and cingulum-sgACC (Cg-sg), and the AN position was determined on the thalamic part of the resulting ANTCF for the MTT fiber tracking (Figure 1A; Choi et al., 2018). Second, for MTT fiber tracking, the AN and middle of the MTT were selected as the seed_point and waypoint_mask, respectively (Figure 1A). From the result of the processing, the fibers which starting from the seed_point AN and passing through waypoint_mask MTT area are generated. We searched for the MTT fiber with a setting of the maximum fiber length of 15 mm. The MTT area was clearly distinguishable from the fornix in the 7-Tesla super-resolution TDI image (see Supplementary Figure S2). The fiber tracking of the cingulum-PHG (Cg-ph) with PP were performed by seed_point in the PHG with waypoint_mask in the hippocampal formation (see Figure 1B). Lastly, for the tracking of the fornix, the body of fornix was selected as the seed_point (Figure 1A).

## Results

**Figure 2:**
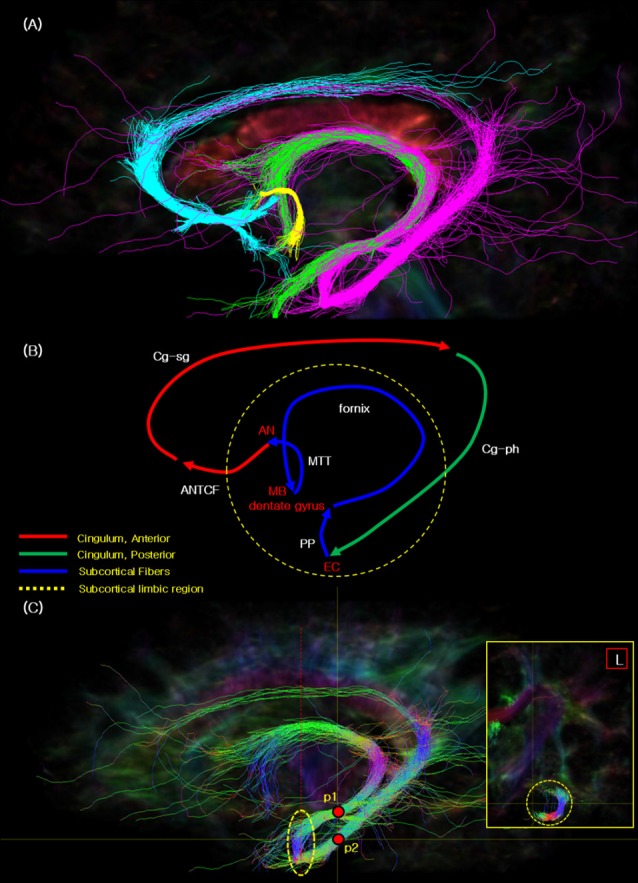
The seed tracking result of the complete Papez circuit. **(A)** The resulted fiber tracks of the Papez circuit showing the cyan: ANTCF, Cg-sg; magenta: Cg-ph, PP area, fornix; green: fornix; yellow: mammillothalamic tract (MTT). **(B)** The resulted flow lines of the Papez circuit showing the red: ANTCF, Cg-sg; green: Cg-ph; blue: PP area, fornix, and MTT. The names of nerve fiber in the image are indicated with the white color, and other landmarks were red. **(C)** The seed tracking result of the PP in the EC area with the directional color. This main sagittal image presents the two seed_points (p1 and p2) with red points, and yellow dot circle which indicate the connectivity areas with the PP. Red dot line in the sagittal image indicate the coronal section line of the small yellow box in the right side. The coronal section image in the right shows the seed tracking results which illustrate the connectivity in the EC. Legend: EC, entorhinal cortex; MB, mammillary body; PP, perforant path.

Figure 2A presents the fiber tracking results of the Papez circuit with different colors labeled for each fiber tracking. Through current study, for the first time, we were able to visualize the entire Papez circuit; that is, ANTCF (cyan), Cg-sg (cyan), Cg-ph (magenta), PP (magenta), fornix (magenta, green), and MTT (yellow; Jones et al., 2013; Choi et al., 2018). Part of cingulum in the sgACC area is abbreviated as Cg-sg while part of cingulum in the PHG area as Cg-ph, respectively. ANTCF is a thalamocortical fiber which connects the AN to the cingulate cortex, and recent study suggested that the ANTCF connect that *via* the septal area as with this result (Choi et al., 2018). Through additional analysis studies from the other subject data, we have confirmed the reproducibility and reliability of the study (see Supplementary Figure S3).

Figure 2B shows the flow path we observed of the Papez circuit which is presented in Figure 2A. Each fiber track is color labeled as, ANTCF (red), Cg-sg (red), Cg-ph (green), PP (blue), fornix (blue), and MTT (blue; compare with the concept image in Supplementary Figure S1 and the result image in Figure 2A. As seen from Figure 2B, cingulum seems separated in two segments, as anterior segment (Cg-sg) and posterior segment (Cg-ph), respectively. Interestingly, the two branches of the cingulum, (Cg-sg and Cg-ph) are also clearly distinguishable in the coronal image of the cingulum (see Supplementary Figure S4, Jones et al., 2013; Wu et al., 2016; Wei et al., 2017). Note there are several studies of functional localization in the cingulum that report that the anterior portion of the cingulate gyrus is functionally motor, while the posterior cingulate is more involved in sensory integration (Vogt et al., 1992). This observation is different from the original Papez’s report which suggested that the Papez circuit is an integrated single closed loop starting from hippocampal formation and back to the cingulate gyrus (Papez, 1937).

Figure 2C presents another interesting observation that the seed tracking result of the EC area with the hippocampal formation and PHG is connected. The EC is interposed between the hippocampal formation (architecortex) and PHG (neocortex). In this complex, the PP connects the EC and the dentate gyrus, thereby, it connects to the hippocampal formation (Lee and Park, 2012). From the dentate gyrus, the PP is connected to the fornix *via* the mossy fiber pathway. Additionally, the PP is also connected to posterior part of the cingulum (Cg-ph), and thereby forms the entire Papez circuit (see Figure 2C). It is, therefore, can be summarized as three parts closed loop connection circuit, that is, anterior cingulate part, posterior cingulate part, and the subcortical limbic region.

The image inserted in the yellow box in the right side of Figure 2C illustrates the “J” shaped connection *via* PP between the dentate gyrus and EC. Previously, both *ex vivo* (Augustinack et al., 2010; Zeineh et al., 2017) and *in vivo* (Yassa et al., 2010; Zeineh et al., 2012) imaging studies of the PP have been conducted, however, majority of studies depict only the local view without providing the global connectional view of the entire Papez circuit as we have observed.

## Discussion and Conclusions

In this study, for the first time, we succeeded in visualizing the entire Papez circuit in the *in vivo* human brain using 7-Tesla super-resolution TDI with track tracing technique. Especially, we have succeeded in visualization of the PP and the ANTCF in the Papez which could not hitherto be visualized. It should be noted that instead of using the indirect structural MRI template, we have located the seed positions directly from the super-resolution 7-Tesla TDI image. This approach, in turn, allowed us to locate the seed positions substantially better than with the structural MRI template. As the contribution of that, we could identify the cingulum to PHG—EC—hippocampal formation pathway for the first time, and we could also determine the seed_point of the MTT and ANTCF more accurately.

Finally, our experimental results demonstrated that the cingulum consists of two distinguishable segments, the anterior segment and posterior segment. This pattern of the cingulum can be correlated with the several previous studies that the ACC is largely involved in cognitive functions on emotional aspect while the posterior aspect is more acting as a sensory integrating aspect (Vogt et al., 1992; Raichle et al., 2001; Shackman et al., 2011). Also, our results show that the fiber connection in the amygdala as parts of the fornix that extend anterior to the perforant pathway. This result is consistent with the recent report which described the amygdalothalamic tract that travels with the fornix (Kamali et al., 2018).

In summary, we succeeded in visualizing the entire fiber components of the Papez circuit in the *in vivo*human brain, ANTCF—(cingulate gyrus)—cingulum—(EC)—PP—(hippocampal formation)—fornix—(MB)—MTT—(AN). We believe that the observation not only supports the neuro anatomy of human memory circuitry, but it also provides new insights for the understanding of the basic mechanism related to memory and cognition.

## Author Contributions

S-HC suggested the fiber, data processing, and draft of the manuscript. S-HP and Y-BK assisted with neuro-anatomical consultation. Z-HC formulated the super-resolution tractography and its application to neural circuitry.

## Conflict of Interest Statement

The authors declare that the research was conducted in the absence of any commercial or financial relationships that could be construed as a potential conflict of interest.
