# Supplementary material for: Papez Circuit Observed by in vivo Human Brain With 7.0T MRI Super-Resolution Track Density Imaging and Track Tracing
Source: Front Neuroanat. 2019 Feb 18;13:17. doi: 10.3389/fnana.2019.00017 (PMC6387901; doi:10.3389/fnana.2019.00017)
Supplement: Supplementary file 1 [file Data_Sheet_1.PDF]

Supplementary Material

Papez circuit observed by in-vivo human brain with 7.0T Super-Resolution MR Track density imaging and track tracing

[1]Sang-Han Choi Ph.D, [2]Yong-Bo Kim MD, Ph.D, [3]Sun-Ha Paek MD, Ph.D, [1,4]Zang-Hee Cho* Ph.D

[1]Neuroscience Research Institute, Suwon University, Gyeonggi, South Korea

[2]Neuroscience Research Institute, Gachon University, Incheon, South Korea

[3]Department of Neurosurgery, Seoul National University Hospital, Seoul, South Korea

[4]AICT, Seoul National University, Seoul, South Korea

*** Correspondence:** Zang-Hee Cho

zcho1@snu.ac.kr, zhcho36@gmail.com

1 Supplementary Figures and Tables

1.1 Supplementary Figures

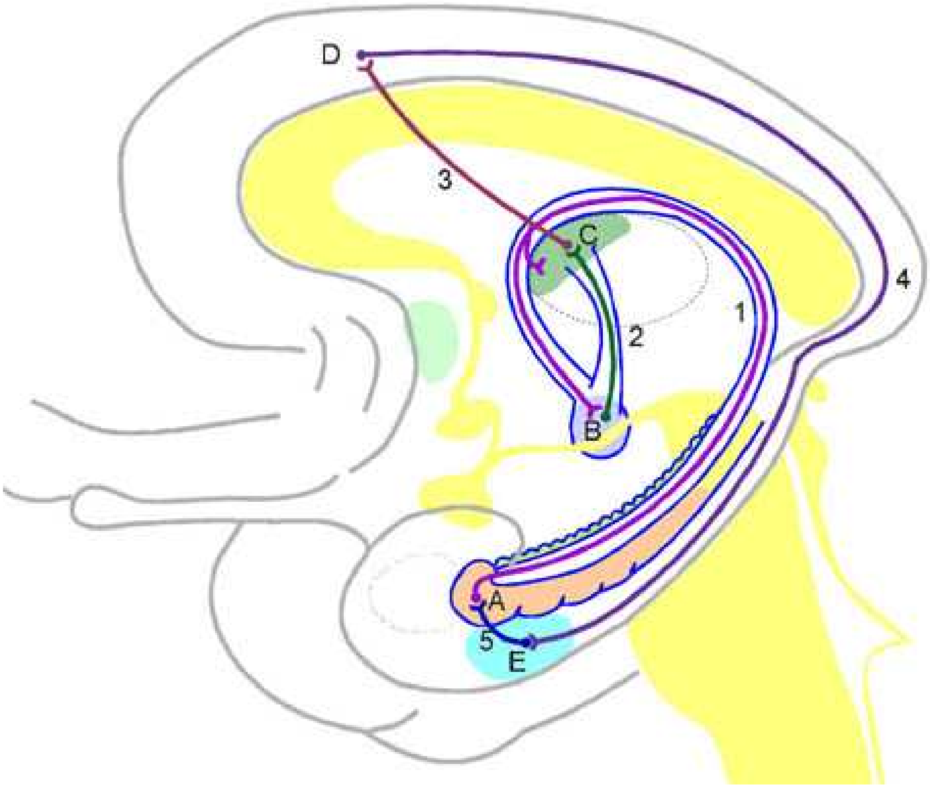

Supplementary Figure 1. The classical Papez circuit mid sagittal view courtesy of LEE WT (Lee and Park, 2008). The index numbers and characters in the image correspond to the following: 1. fornix, 2. mammillothalamic tract, 3. thalamocortical fiber of the anterior nucleus, 4. cingulum, 5. perforant path, A. hippocampal formation, B. mammillary body, C. anterior nucleus of thalamus, D. cingulate gyrus, and E. entorhinal cortex.

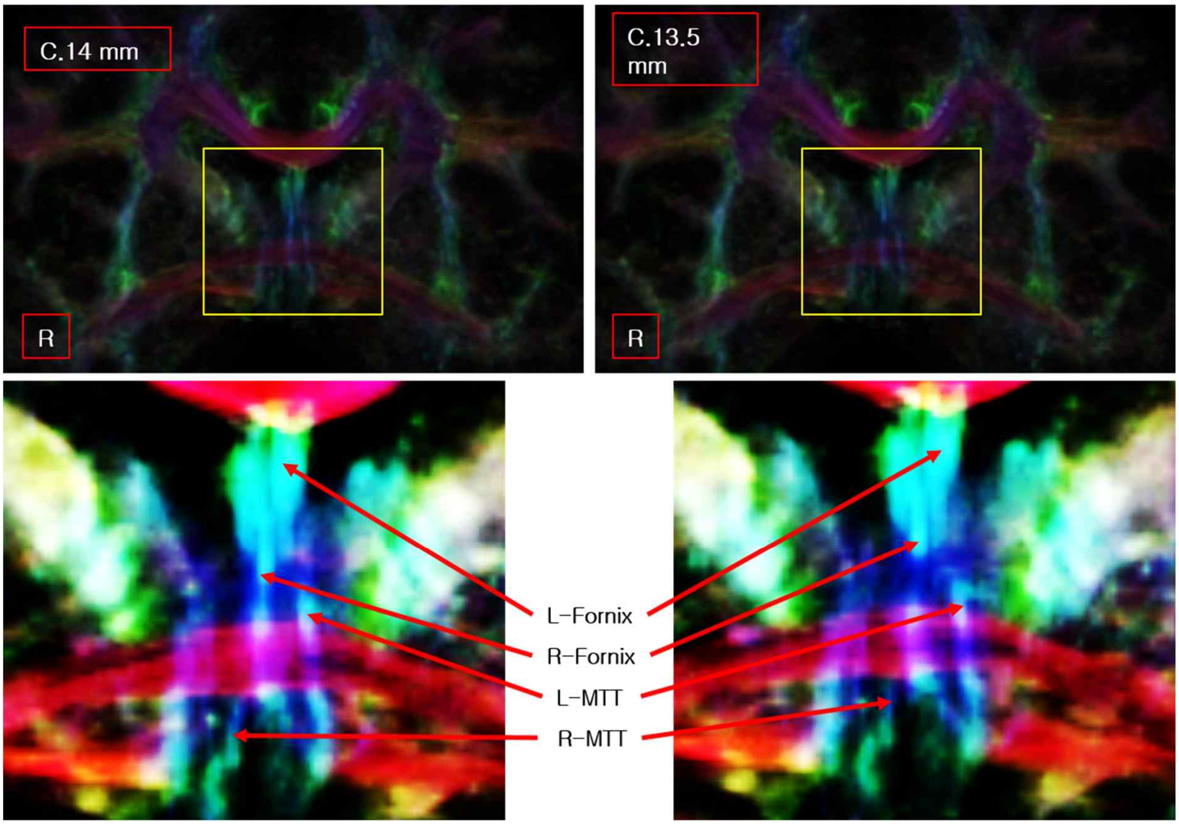

Supplementary Figure 2. The position of the MTT from the TDI coronal image. Left column: coronal 14 mm, right column: coronal 13.5 mm, bottom row: size- and contrast-controlled image of the yellow box in each top image.

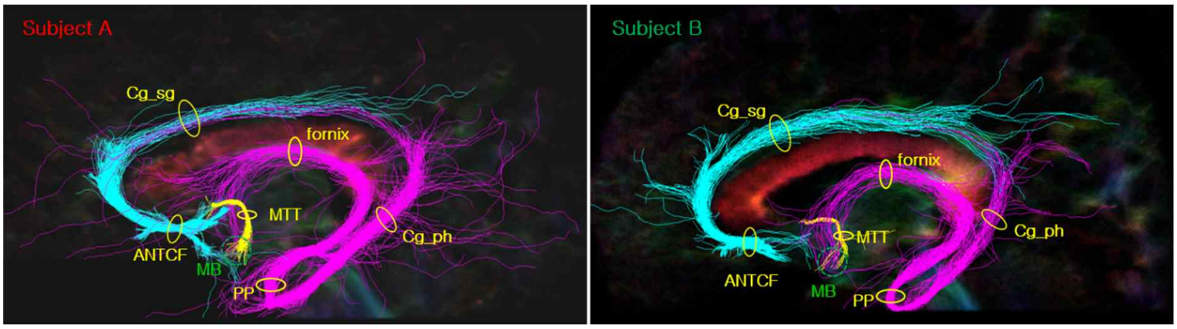

Supplementary Figure 3. The seed tracking result of the entire Papez circuit from two subjects with the labelled color. ANTCF (cyan), MTT (yellow), PP area (magenta). MB: mammillary body, PP: perforant path

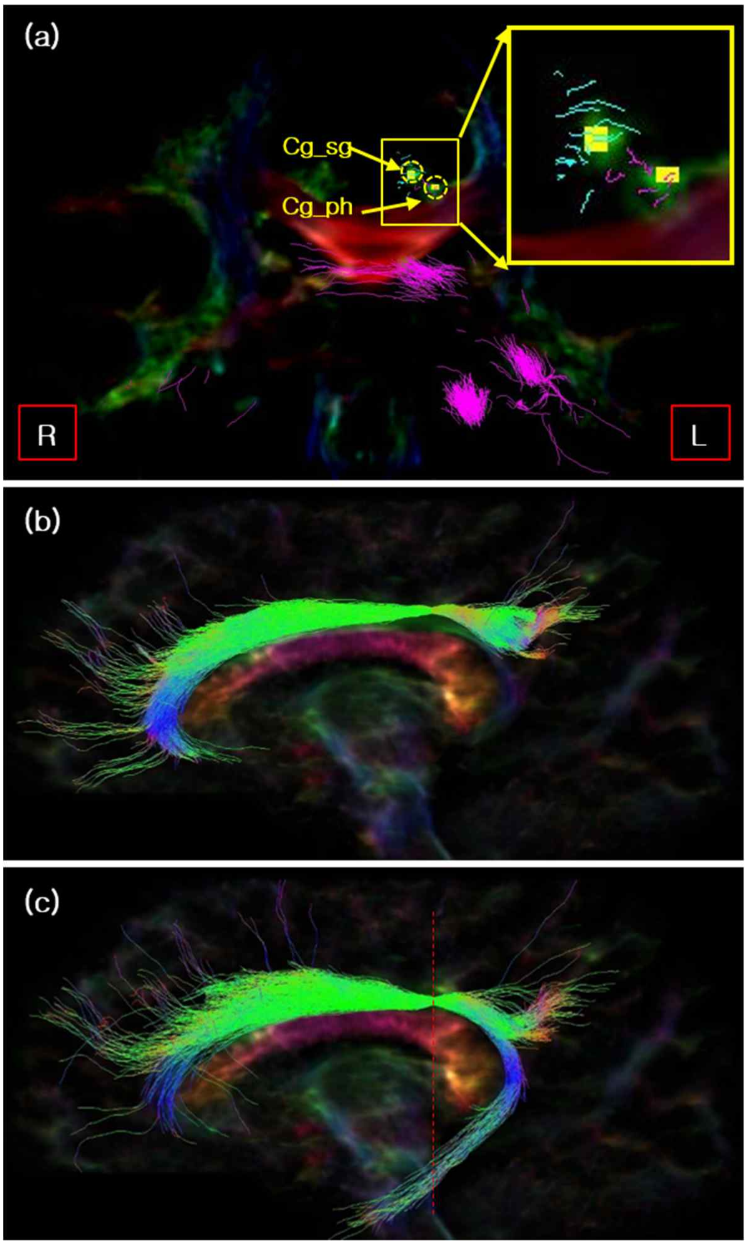

Supplementary Figure 4. The subdivision of the cingulum in the coronal view (-16mm), and the fiber tracking results. (A) The Cg-sg (upper cingulum, cyan) and Cg-ph (lower cingulum, magenta) seeds, and their magnified image (yellow box), see Fig. 3. (B and C) Sagittal image of the seed tracking results of the Cg-sg (B) and Cg-ph (C). The red dotted line in image (C) indicates the seed slice cut.
